# Effects of animal-assisted psychotherapy incorporating mindfulness and self-compassion in neurorehabilitation: a randomized controlled feasibility trial

**DOI:** 10.1038/s41598-022-14584-1

**Published:** 2022-06-28

**Authors:** Pascale Künzi, Michael Ackert, Martin grosse Holtforth, Margret Hund-Georgiadis, Karin Hediger

**Affiliations:** 1 Division of Clinical Psychology and Psychotherapy, Faculty of Psychology, University of Bern, Bern, Switzerland; 2 REHAB Basel, Clinic for Neurorehabilitation and Paraplegiology, Basel, Switzerland; 3 Psychosomatic Medicine, Department of Neurology, Inselspital, Bern University Hospital, Bern, Switzerland; 4 Division of Clinical Psychology and Animal-Assisted Interventions, Faculty of Psychology, University of Basel, Basel, Switzerland; 5 Department of Epidemiology and Public Health, Human and Animal Health Unit, Swiss Tropical and Public Health Institute, Allschwil, Switzerland; 6 Institute for Interdisciplinary Research on Human-Animal Interaction (IEMT), Allschwil, Switzerland; 7 Department of Psychology, University of Fribourg, Fribourg, Switzerland; 8 Faculty of Psychology, Open University, Heerlen, The Netherlands

**Keywords:** Brain injuries, Anxiety, Depression, Rehabilitation

## Abstract

Transdiagnostic psychotherapeutic approaches are increasingly used in neurorehabilitation to address psychological distress. Animal-assistance is thought to increase efficacy. The present study evaluates a psychotherapeutic mindfulness- and self-compassion-based group intervention (MSCBI) with and without animal-assistance for patients with acquired brain injury. Patients (*N* = 31) were randomly assigned to the 6-week intervention with (*n* = 14) or without animal-assistance (*n* = 17). Primary outcome was psychological distress at post- and follow-up treatment, secondary outcomes were changes within-session of patients’ emotional states, adherence to treatment and attrition. Psychological distress significantly decreased in both groups from pre- to follow-up treatment with no difference between groups. Patients in the animal-assisted MSCBI group reported significantly higher increases in feeling secure, accepted, comforted, grateful, motivated and at ease during the sessions compared to patients in the MSCBI group without animal-assistance. Adherence to sessions was significantly higher in the animal-assisted MSCBI group. Attrition did not significantly differ between groups. Our results show that both MSCBIs with and without animal-assistance are feasible and effective in reducing psychological distress in patients with acquired brain injury. The significant changes within-sessions mainly in relationship-based emotional states and the higher treatment adherence suggest additional effects of animal-assistance. Animal-assistance might increase acceptability and patients’ commitment to psychotherapy.

## Introduction

Acquired brain injury of traumatic or non-traumatic origins is a globally significant public health issue. Incidence rates for traumatic brain injury in Europe vary between 47 to 649 per 100,000 population per year, affecting approximately 50–60 million people worldwide^1,2^. Psychiatric disorders often co-occur^3^ with an accumulation of depression^4^ and post-traumatic stress disorder^5^. While the 3-month prevalence for depression following traumatic brain injury is 56%^6^, the long-term prevalence for depression is estimated at 43% and for anxiety disorders at 36%^7^. Comorbidity of both depression and anxiety is associated with a negative impact on rehabilitation^8^ and a poorer health-related quality of life^9^. A particularly critical period for the emergence of psychiatric disorders is the first year following traumatic brain injury^10^. It is therefore crucial to provide early psychotherapeutic treatment for patients with acquired brain injury to improve their long-term outcomes of rehabilitation^11^.

Although still sparsely represented, psychotherapeutic interventions for patients with acquired brain injury have emerged in the last decade^12–15^. Effective approaches include positive psychotherapy^13^, integrative neuro-psychotherapy incorporating interventions of cognitive behavioral therapy^12^, acceptance and commitment therapy^14^ as well as compassion-focused therapy^15^. Research shows that psychotherapy can help to ameliorate the adjustment-process to acquired brain injury^16^ and promising evidence summarizes the effectiveness of psychological interventions for patients with acquired brain injury on depressive symptoms with an overall medium effect size of *d* = 0.69 when compared to control conditions^17^. The concept of self-compassion holds especially promising capacities^18^ and is defined as “a compassion directed inward, relating to ourselves as the object of care and concern when faced with the experience of suffering”^19^. A lack of self-compassion and increased levels of self-criticism have been associated with the development and maintenance of a range of psychological disorders^20^. A meta-analysis found a large effect size for the relationship between self-compassion and psychopathology^21^. Self-compassion-based interventions play an important role in gaining strength and resilience when faced with life stressors such as chronic health issues^22^. As defined by Neff and Dahm^19^, one core element of self-compassion is mindfulness. Practicing mindfulness shows beneficial effects for patients with acquired brain injury regarding overall symptom load, mental health and quality of life^23^, as well as in perceived self-efficacy^24^. It is also associated with a reduction in symptoms of post-traumatic stress^25^, depression^26^ and mental fatigue^27–29^. A recent review concludes that such transdiagnostic psychotherapeutic interventions for patients with neurological conditions can lead to a significant overall reduction in emotional distress, although some studies found mixed results^30^. However, mindfulness- and self-compassion-based interventions are usually challenged by low adherence to treatment and high attrition rates, ranging from 13% up to 61% for patients with acquired brain injury^26,28,31–33^. Therefore, effective and attractive psychotherapeutic interventions for patients in neurorehabilitation are urgently needed.

One method that is currently discussed to increase acceptability and effectiveness of psychotherapeutic interventions is animal-assistance^34^. An animal-assisted mindfulness intervention for patients with recurrent depression was found to lead to a decrease in depressive symptoms and rumination, and an improvement in overall mindfulness skills with no dropouts^35^. Other studies indicate positive effects of interacting with animals on social behavior and motivation^36,37^, mood^38^, establishing contact, communication and relaxation^39^, stress-related parameters and positive emotional-physiologic states^40^, anxiety^41,42^, episodic memory^43^, concentration^44,45^, and engagement in behavioral and mental health services^34^. Integrating a domesticated animal in a psychotherapeutic intervention, delivered by a psychotherapist with additional certification in animal-assisted therapy, is conceptualized as animal-assisted psychotherapy^46,47^. Conducted in a highly relational environment, animal-assisted psychotherapy is discussed to be an approach that lowers barriers to utilization of psychotherapeutic interventions^48^ by enriching the therapeutic setting through a context of normality and non-evaluation which helps patients to feel secure and accepted^49,50^. Animals are hypothesized to act as social-support figures^51^. Therefore animal-assistance might enhance the acceptance and completion of psychotherapeutic interventions. Animal-assistance is also associated with enhanced perceptions of the psychotherapist regarding trustworthiness and an increased willingness to disclose^52^. Alliance ruptures in psychotherapy may occur with either the therapist or the animal, but usually not with both^49^. Studies show that integrating an animal into psychotherapy can lead to higher treatment adherence^53^, higher ratings of therapist efficacy and willingness to participate in future mindfulness trainings for clients experiencing psychological distress^54^. Although first evidence regarding the benefit of animal-assistance in psychotherapy exists, research of differences in application and modes of action regarding animal-assistance is limited.

To close this gap, the present study investigates the feasibility of an injury-adapted psychotherapeutic mindfulness- and self-compassion-based group intervention (MSCBI) for patients with acquired brain injury in inpatient neurorehabilitation. Groups with (animal-assisted MSCBI) and without animal-assistance (standard MSCBI) are compared. We evaluate its effects on the patients’ general psychological distress at post- and follow-up treatment as well as changes in emotional states within-sessions, adherence to treatment and attrition.

## Results

### Sample characteristics

**Figure 1:**
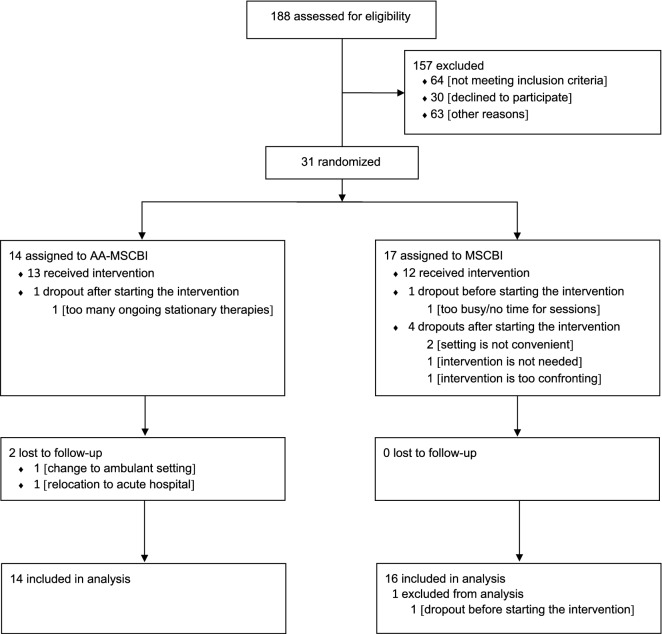
Patients flowchart for the animal-assisted psychotherapeutic mindfulness- and self-compassion-based group intervention (AA-MSCBI) and standard psychotherapeutic mindfulness- and self-compassion-based group intervention (MSCBI).

Table 1 shows the baseline demographics and sample characteristics for the patients in the animal-assisted and the standard MSCBI group. One patient in the standard MSCBI group dropped out after randomization but before starting the intervention and therefore was excluded from the analyses. This led to a final sample size of *N* = 30 patients (animal-assisted MSCBI: *n* = 14; standard MSCBI: *n* = 16, see flowchart, Fig. 1). Regarding the primary outcome, twenty-five patients attended at least 9 of 12 sessions and were classified as completers (80.6%; animal-assisted MSCBI: *n* = 13; standard MSCBI: *n* = 12). The analyses of in-session changes of patients’ emotional states were performed with all patients attending the given session allocated to either the animal-assisted MSCBI group (*n* = 14) or the standard MSCBI group (*n* = 16). As some patients did not attend all of the planned sessions, we collected 292 pre-session scores and 290 post-session scores, leading to a total of 290 analyzed change scores (out of 360 potential change scores, see Table 3). The two groups did not differ regarding demographic variables and baseline measurements besides affection to animals which was higher in the animal-assisted MSCBI (see Table 1).

**Table 1:** Baseline demographics and sample characteristics.

	AA-MSCBI	MSCBI	Statistics
	*N* = 14	*N* = *17*	
Age in years, *M* (range)	42.36 (26 – 66 years)	45.47 (27 – 64 years)	*Difference* = 3.17, CI − 5.82 to 12.17, *p* = 0.476
**Gender, *N* (%)**			
Male	9 (64.30)	12 (70.60)	*Difference* = 1.33, CI 0.29 to 6.04, *p* = 1.000
Female	5 (35.70)	5 (29.40)	
**Marital status, *N* (%)**			
Single/living alone	8 (57.10)	12 (70.60)	*Difference* = 1.80, CI 0.41 to 7.96, *p* = 0.477
Married/living together	6 (42.90)	5 (29.40)	
**Highest education, *N* (%)**			
Basic (compulsory and secondary school/apprenticeship)	6 (42.90)	13 (76.50)	*Difference* = 4.33, CI 0.93 to 20.24, *p* = 0.075
Secondary (college/university)	8 (57.10)	4 (23.50)	
**Premorbid psychological difficulties, *N* (%)**			
Yes	4 (28.60)	6 (37.50)	*Difference* = 1.50, CI 0.32 to 6.99, *p* = 0.709
No	10 (71.40)	10 (62.50)	
Not specified: missing^a^	0	1	
**Psychological treatment,* N* (%)**			
Current	8 (61.50)	7 (50.00)	*Difference* = 0.63, CI 0.14 to 2.89, *p* = 0.547
Past/current and past	5 (38.50)	7 (50.00)	
Not specified: missing^a^	1	3	
**Rehabilitation setting, *N* (%)**			
Residential	9 (64.30)	8 (47.10)	*Difference* = 0.49, CI 0.12 to 2.11, *p* = 0.337
Semiresidential/ambulant	5 (35.70)	9 (52.90)	
**Diagnosis, *N* (%)**			
TBI	5 (35.70)	5 (29.40)	*Difference* = 0.75, CI 0.17 to 3.41, *p* = 1.000
Non-TBI	9 (64.30)	12 (70.60)	
**Time** **since** **injury** **(months),** ***M (SD)***	5.21 (5.79)	6.20 (5.67)	*Difference* = 1.29, CI − 2.98 to 5.56, *p* = 0.542
**Cognitive impairment**			
MoCA *M (SD)*	25.57 (2.68)	24.27 (2.60)	*Difference* = 1.41, CI − 0.49 to 3.28, *p* = 0.142
**Psychological characteristics**			
GSI pre-treatment *M (SD)*	0.71 (0.65)	0.63 (0.80)	*Difference* = 0.08, CI − 0.48 to 0.63, *p* = 0.782
Affection to animals *M (SD)*	5.86 (0.36)	4.75 (1.07)	*Difference* = 1.11, CI 0.51 to 1.70, *p* = 0.001**
**Owner of a pet *N* (%)**			
Yes	7 (50.00)	7 (41.20)	*Difference* = 0.70, CI 0.17 to 2.91, *p* = 0.623
No	7 (50.00)	10 (58.80)	

*AA-MSCBI* animal-assisted psychotherapeutic mindfulness- and self-compassion-based group intervention, *MSCBI* standard psychotherapeutic mindfulness- and self-compassion-based group intervention, *N* number of patients, % percentage of patients,* TBI* traumatic brain injury,* Non-TBI* non-traumatic brain injury, *M* mean, *SD* standard deviation, *MoCA* Montreal Cognitive Assessment,* GSI* Global Severity Index of the Brief Symptom Inventory reflecting patients’ general psychological distress, ^a^: not included in analysis, **p < 0.01.

### Effects on general psychological distress

We found a significant decrease over time for the Global Severity Index score in both groups regarding the pre- to follow-up time span (t_1_–t_3_: *Difference* = − 0.29, CI − 0.54 to 0.03, *p* = 0.028) with a down-trend from pre- to post-treatment (t_1_–t_2_: *Difference* = − 0.22, CI − 0.43 to 0.00, *p* = 0.051). We found no significant difference between groups nor an interaction effect (group: *Difference* = 0.16, CI − 0.15 to 0.47, *p* = 0.289; group*t_1_–t_2_: *Difference* = 0.18, CI − 0.26 to 0.61, *p* = 0.412; group*t_1_–t_3_: *Difference* = 0.09, CI − 0.42 to 0.59, *p* = 0.772), indicating no significant difference between the animal-assisted MSCBI group and the standard MSCBI group regarding reduction in the patients’ general psychological distress (see Table 2).

**Table 2:** General psychological distress.

Group	Timepoint	GSI	
		*N*	*M *(*SD*)
AA-MSCBI	Pre-treatment	14	0.71 (0.65)
	Post-treatment	14	0.58 (0.51)
	Follow-up treatment	14	0.46 (0.33)
MSCBI	Pre-treatment	16	0.63 (0.80)
	Post-treatment	16	0.33 (0.32)
	Follow-up treatment	16	0.30 (0.29)
Total	Pre-treatment	30	0.67 (0.72)
	Post-treatment	30	0.45 (0.43)
	Follow-up treatment	30	0.38 (0.31)

*AA-MSCBI* animal-assisted psychotherapeutic mindfulness- and self-compassion-based group intervention,* MSCBI* standard psychotherapeutic mindfulness- and self-compassion-based group intervention,* GSI* Global Severity Index of the Brief Symptom Inventory reflecting patients’ general psychological distress, *N* number of patients, *M* mean, *SD* standard deviation.

### Effects on emotional states (pre- to post-session)

#### Secure

The groups significantly differed regarding their within-session change in feeling secure (*Difference* = 17.78, CI 7.78 to 27.78, *p* = 0.001). We found a significant increase in the Visual Analogue Scale (VAS) ﻿variable *secure* in the animal-assisted MSCBI group, indicating that patients felt more secure after the sessions (*Difference* = 18.69, CI 9.92 to 27.26, *p* < 0.001). In the standard MSCBI group there was no significant increase from pre- to post-sessions in the VAS variable *secure* (*Difference* = 1.51, CI − 13.58 to 16.61, *p* = 0.844). The increase in feeling secure for patients in the animal-assisted MSCBI group was 12-times higher when compared to the standard MSCBI group. Session number did not have an effect, indicating that the increase in feeling secure after the sessions did not change over time (see Table 3 and Figure 1S in Appendix 1).

**Table 3:** Change in patients’ emotional states from pre- to post-sessions within-groups, between-groups and over time (session 1–12) for the animal-assisted psychotherapeutic mindfulness- and self-compassion-based group intervention (AA-MSCBI) and the standard psychotherapeutic mindfulness- and self-compassion-based group intervention (MSCBI).

VAS	AA-MSCBI change score *N* = 151	MSCBI change score *N* = 139	Group-Level (AA-MSCBI vs MSCBI)			Session-Level (from session 1–12)		
			*Difference*	95% CI	*p*-value	*Difference*	95% CI	*p*-value
	*M* (*SD*)	*M* (*SD*)						
Secure	19.42 (33.41)	1.41 (19.55)	17.78	7.78 to 27.78	0.001**	0.20	− 0.67 to 1.07	0.645
Comforted	11.90 (27.23)	− 0.32 (21.07)	13.23	4.50 to 21.96	0.003**	0.05	− 0.72 to 0.83	0.892
Accepted	12.64 (27.09)	2.31 (14.80)	10.46	2.87 to 18.06	0.007**	0.23	− 0.47 to 0.93	0.525
Hopeful	8.05 (26.69)	4.52 (29.29)	3.68	− 3.71 to 11.07	0.328	0.00	− 0.92 to 0.93	0.996
Motivated	12.87 (23.48)	1.66 (27.94)	11.33	1.55 to 21.12	0.023*	0.94	0.15 to 1.73	0.021*
Grateful	12.18 (23.50)	4.91 (19.95)	8.02	0.55 to 15.49	0.035*	0.11	− 0.60 to 0.79	0.790
At ease	22.52 (31.61)	2.15 (23.44)	21.41	11.52 to 31.27	< 0.001**	0.82	− 0.07 to 1.70	0.071

*VAS* Visual Analogue Scale, *N* number of change scores, *M* mean, *SD* standard deviation,* 95% CI* 95% confidence interval, *Difference*: coefficient estimating the mean difference as effect size, *p < 0.05, **p < 0.01. A positive change score indicates an increase in the mentioned emotional state from pre- to post-session, a negative change score indicates a reduction in the mentioned emotional state from pre- to post-session.

#### Comforted

The groups significantly differed regarding their within-session change in feeling comforted (*Difference* = 13.23, CI 4.50 to 21.96, *p* = 0.003). Patients in the animal-assisted MSCBI group showed a significant increase in the VAS variable *comforted*, indicating the patients felt more comforted after the sessions (*Difference* = 11.73, CI 5.27 to 18.20, *p* < 0.001). We found no significant increase from pre- to post-sessions in the VAS variable *comforted* in the standard MSCBI group (*Difference* = 1.38, CI − 11.61 to 14.35, *p* = 0.835). The increase in feeling comforted for patients in the animal-assisted MSCBI was 9-times higher when compared to the standard MSCBI group. Session number did not have an effect, indicating that this increase in feeling comforted after the sessions did not change over time (see Table 3 and Figure 2S in Appendix 1).

#### Accepted

The groups did significantly differ regarding their within-session change in feeling accepted (*Difference* = 10.46, CI 2.87 to 18.06, *p* = 0.007). The animal-assisted MSCBI group showed a significant increase in the VAS variable *accepted,* indicating the patients felt more accepted after the sessions (*Difference* = 12.39, CI 5.93 to 18.84, *p* < 0.001). We found no significant increase from pre- to post-sessions in the VAS variable *accepted* in the standard MSCBI group (*Difference* = 1.05, CI − 12.76 to 14.86, *p* = 0.882). The increase in feeling accepted for patients in the animal-assisted MSCBI was 12-times higher when compared to the standard MSCBI group. Session number did not have an effect, indicating that this increase in feeling accepted after the sessions did not change over time (see Table 3 and Figure 3S in Appendix 1).

#### Hopeful

The groups did not significantly differ regarding their within-session change in feeling hopeful (*Difference* = 3.68, CI − 3.71 to 11.07, *p* = 0.328). In the animal-assisted MSCBI group we found a significant increase in the VAS variable *hopeful,* indicating the patients felt more hopeful after the sessions (*Difference* = 8.28, CI 3.92 to 12.65, *p* < 0.001). In the standard MSCBI group there was no significant increase from pre- to post-sessions in the VAS variable *hopeful* (*Difference* = 7.89, CI − 7.60 to 23.38, *p* = 0.318). Session number did not have an effect (see Table 3 and Figure 4S in Appendix 1).

#### Motivated

The groups significantly differed regarding their within-session change in feeling motivated (*Difference* = 11.33, CI 1.55 to 21.12, *p* = 0.023). We found a significant increase in the VAS variable *motivated* during the sessions in the animal-assisted MSCBI group, indicating the patients felt more motivated after the sessions (*Difference* = 13.14, CI 6.76 to 19.52, *p* < 0.001). There was no significant increase from pre- to post-sessions in the VAS variable *motivated* in the standard MSCBI group (*Difference* = 12.09, CI − 3.75 to 27.93, *p* = 0.135). Session number had a significant influence (*Difference* = 0.94, CI 0.15 to 1.73, *p* = 0.021), indicating that the increase in feeling motivated after the sessions became bigger over time (see Table 3 and Figure 5S in Appendix 1).

#### Grateful

The groups significantly differed regarding their within-session change in feeling grateful (*Difference* = 8.02, CI 0.55 to 15.49, *p* = 0.035). The animal-assisted MSCBI group showed a significant increase in the VAS variable *grateful* during the sessions, indicating the patients felt more grateful after the sessions (*Difference* = 12.06, CI 6.42 to 17.70, *p* < 0.001). In the standard MSCBI there was no significant increase from pre- to post-sessions in the VAS variable *grateful* (*Difference* = 9.04, CI − 2.47 to 20.55, *p* = 0.124). Session number did not have an effect, indicating that this increase in feeling grateful from pre- to post-session did not change over time (see Table 3 and Figure 6S in Appendix 1).

#### At ease

The groups significantly differed regarding their within-session change in feeling at ease (*Difference* = 21.41, CI 11.52 to 31.27, *p* < 0.001). The animal-assisted MSCBI group showed a significant increase in the VAS variable *at ease* during the sessions, indicating the patients felt more at ease after the sessions (*Difference* = 22.18, CI 15.05 to 29.31, *p* < 0.001). We found no significant increase from pre- to post-sessions in the VAS variable *at ease* in the standard MSCBI group (*Difference* = 10.02, CI − 13.26 to 33.31, *p* = 0.399). The increase in feeling at ease during the sessions of patients in the animal-assisted MSCBI group was twice as high compared to the patients in the standard MSCBI. Session number did not have an effect, indicating that the increase in feeling at ease from pre- to post-session did not change over time (see Table 3 and Figure 7S in Appendix 1).

### Feasibility and acceptability

#### Adherence to treatment

Patients in the animal-assisted MSCBI group attended 151 of 168 sessions (89.9% completed, 10.1% missed). In the standard MSCBI group, the attendance rate was in total 140 of 192 sessions (72.9% completed, 27.1% missed). The groups differed significantly in their adherence rate (*Difference* = 3.28, CI 1.82 to 5.98,* p* < 0.001) when considering all patients allocated to treatment (see Table 4).

**Table 4:** Adherence to treatment and attrition for the animal-assisted psychotherapeutic mindfulness- and self-compassion-based group intervention (AA-MSCBI) and the standard psychotherapeutic mindfulness- and self-compassion-based group intervention (MSCBI).

	AA-MSCBI	MSCBI	Statistics
Adherence to sessions ^a^, *N* (%)	*N* = 14	*N* = 17	
Attended	151 (89.9)	140 (72.9)	*Difference* = 3.28, CI 1.82 to 5.98,* p* < 0.001**
Missed	17 (10.1)	52 (27.1)	

Attrition, *N* (%)	AA-MSCBI	MSCBI	Statistics
**Before start of the intervention**	*N* = 14	*N* = 17	
Dropout	0	1 (5.9)	*Difference* = 0.53, CI 0.38 to 0.53, *p* = 1.000
Starter	14 (100)	16 (94.1)	

**Intervention period**	*N* = 14	*N* = 16	Statistics
Dropout	1 (7.1)	4 (23.5)	*Difference* = 0.25, CI 0.03 to 2.55, *p* = 0.334
Completer	13 (92.9)	12 (76.5)	

**Total**	*N* = 14	*N* = 17	Statistics
Dropout	1 (7.1)	5 (29.4)	*Difference* = 0.19, CI 0.02 to 1.82, *p* = 0.185
Completer	13 (92.9)	12 (70.6)	

^a^All patients allocated to treatment, *N* number of patients, % percentage of patients, *Difference*: coefficient estimating the mean difference as effect size,* CI* 95% confidence interval, **p < 0.01.

#### Attrition

One patient in the standard MSCBI group dropped out after randomization and before the start of the intervention. In the standard MSCBI group, four patients (23.5%) decided to discontinue the intervention whereas in the animal-assisted MSCBI group one patient (7.1%) stopped the intervention. These five patients were classified as dropouts (see Table 4). There were no statistically significant differences on any measured patient characteristics at pre-treatment between completers and dropouts (all *p*’s > 0.118). Reasons for dropping out of the study pre- and during treatment are specified in Fig. 1. The groups did not differ statistically regarding their attrition rate (*Difference* = 0.19, CI 0.02 to 1.82, *p* = 0.185).

#### Adherence to protocol

In all sessions, 100% of the required elements were covered by the therapists in both treatment groups.

## Discussion

We found a significant decrease in patients’ general psychological distress over both groups from pre- to follow-up treatment with a trend from pre- to post-treatment. These results provide evidence for the effectiveness of the MSCBI. Our finding is in line with several studies showing that transdiagnostic treatment approaches significantly reduce emotional distress for patients with neurological conditions^23,30^. Moreover, our result indicates that the adapted, shortened, and intense MSCBI for patients with acquired brain injury is feasible and that this population can benefit from the injury-adjusted format. We found no differences between the groups with and without animal-assistance in the reduction in patients’ general psychological distress. This is in contrast to the results of previous studies with different populations that found short-term effects of animal-assistance on anxiety^41,42^ and on stress-related parameters such as a decrease in heart rate, increase in heart rate variability, increase in salivary oxytocin, and subsequent tympanic membrane temperature changes^40^. Therefore, we expected animal-assistance to have a beneficial longer-term effect reflected in a greater reduction in the patients’ general psychological distress when compared to the standard MSCBI group. However, our data could not confirm this hypothesis thus leading to the conclusion that animal-assistance does not yield differential long-term effects. Our active control group (standard MSCBI) has possibly made it harder to detect a significant difference between groups. Active control interventions are usually expected to produce beneficial effects regardless of their specifc contents^55^. This explanation is in line with an earlier study comparing a modified MBSR-intervention with and without animal-assistance for clients experiencing psychological distress where all participants experienced fewer anxiety and depressive symptoms, decreased psychological distress, and increased mindfulness skills from pre- to post-treatment with no significant difference between groups^54^.

Regarding the within-session change in patients’ emotional states we found significant differences in the mainly relationship-based semantic differentials between the animal-assisted versus standard MSCBI group. The patients in the animal-assisted MSCBI group had a 12-fold higher increase in feeling secure and feeling accepted during the sessions when compared to the standard MSCBI group. Moreover, animal-assistance enlarged the increase in feeling comforted by a factor of nine and the increase in feeling at ease by a factor of two. Animal-assistance evidenced a slightly higher increase in feeling grateful and motivated. These results are in line with a study showing that patients with acquired brain injury show more positive emotions and experience better mood, a higher treatment motivation and satisfaction during therapy sessions in the presence of an animal compared to standard therapy sessions^36^. In another study, the authors showed that dog-assisted therapy for children with severe neurological impairments predominantly leads to experiencing fun, establishing contact, communication and relaxation^39^. Animal-presence seems to also enhance emotional involvement of patients in a minimally conscious state^56,57^. Altogether, our results show that animal-assistance in psychotherapy can have substantial process-based effects within-sessions. For the group with animal-assistance, the increases in feeling secure, accepted and comforted are thought to have an important impact on the therapeutic alliance in psychotherapy.

Adherence to treatment was significantly higher in the animal-assisted MSCBI group when compared to the standard MSCBI group when considering all patients allocated to treatment. The attrition rate did not differ significantly in both groups, although it was lower in the animal-assisted MSCBI group. These results complement findings of a study comparing a dialectic behavioral therapy (DBT) skills group for incarcerated women with self-harm histories. In this study, the group with animal-assistance had a significantly higher adherence to treatment and less dropouts compared to the group without animal-assistance^58^. However, a recent systematic review and meta-analysis for post-traumatic stress disorder found animal-assisted and standard interventions to be equivalent with regard to dropouts^59^. The low adherence to treatment and high attrition rates in mindfulness- and self-compassion-based interventions in general are relatively unexplored fields, highlighting the importance for further investigation^60^. Animal-assistance is hypothesized to be one possible approach to address these challenges. The adherence- and attrition rates of different groups of patients might provide information for differential indication regarding animal-assistance in psychotherapy.

The beneficial effects of animal-assistance on patients’ mainly relationship-based emotional states during the sessions might explain the higher treatment adherence. These results underline one of the most consistent findings in psychotherapy research: the importance of a strong therapeutic alliance for a better treatment outcome^61^, particularly in group therapy^62^. Clients’ decisions to prematurely terminate treatment may depend more on common factors than on the specific type of treatment being used, leading to the important association between the therapeutic alliance and dropouts^63^. Taken together, animal-assistance might be a seminal approach for the psychotherapy of patients with acquired brain injury particularly supporting the therapeutic alliance between patient and therapist.

## Limitations, strengths and future directions

Due to the relatively small sample size and the rather heterogenous sample, the results regarding our primary outcome must be interpreted carefully. Patients and therapists could not be blinded because animals were either present or not. Additionally, our results do not reflect the effect of the relationship to one specific animal in a psychotherapeutic context, but rather the effect of the integration of animals into psychotherapy per se. Patients interacted with several different animals, which on one hand made it harder to build a stable relationship between patient and animal and on the other hand increased the probability that each patient found a “favorite” animal. The difference in the patient’s affection to animals that we found between the two groups could have affected our findings. Since we found effects of animal-assistance on within-session changes on patients’ emotional states and on adherence to sessions, it is hypothesized that the level of affection to animals might influence the effect of animal-assistance and could therefore be an important predictor for effects of animal-assisted programs. However, we did not investigate this hypothesis in our study and suggest to increase the range in patient’s affection to animals and to systematically control it to assess its influence on outcomes in future trials. We did not measure adherence to home practice in this study. Thus, we cannot discuss the importance of this factor as a potential moderator. We suggest that future research assesses patients’ adherence to home practice. Since we adapted the treatment protocol to the special needs of patients with acquired brain injury and combined different methods for the evaluated MSCBI, the intervention cannot be directly compared to other mindfulness- and self-compassion-based programs. This, however, is also a strength of the study. The treatment protocol accounted for the problems of the patients with acquired brain injury by highly structuring all the sessions, delivering simplified therapy materials with illustrations and memory cards, and tools for integrating the learned contents into daily life. It is important to evaluate psychotherapeutic interventions for this patient group who have a high risk for developing psychological disorders. The intervention was manualized and therapist’s adherence to the treatment protocol was assessed. In a rigorous trial design, we compared two active intervention groups with and without animal-assistance which allowed us to evaluate the MSCBI regarding reduction in general psychological distress and also enabled us to investigate effects of animal-assistance. We controlled for therapist allegiance regarding animal-assistance by involving different psychotherapists for both groups and stratified patients regarding their age and cognitive status.

In future research, our findings should be replicated with bigger sample sizes. Future trials should also include a waiting list control group with later access to treatment to control for spontaneous remission which cannot be excluded with our study design. Additionally, we suggest future research to increase the range in patient’s affection to animals to draw more detailed conclusions regarding the differential indication for animal-assistance in psychotherapy.

Considering the significant within-sessions changes of patients’ emotional states, the question arises how these findings can be incorporated into future trials and in the further development of animal-assisted psychotherapy. Future trials should investigate the effects of animal-assistance within mindfulness- and self-compassion-based programs further and include different outcome measurements to make sure we understand the full potential but also the limitations of this approach. Of special relevance in the context of cost-effectiveness will be the question for whom animal-assistance in a therapeutic context might be especially helpful. Previous research already showed that some patients with acquired brain injury profit more from the animal’s presence than others^36^. Animal-assisted psychotherapy is thought to be especially helpful for patients experiencing difficulties in interpersonal relationships^64,65^ and survivors of developmental trauma^48^. Further studies should focus on process- and relationship-based measures to better understand effects of animal-assistance on the therapeutic alliance. Aspects of forming a relationship of patients with complex trauma with a specific therapist with and without animal-assistance should be investigated, as well as handling of alliance ruptures and attrition in psychotherapies with and without animal-assistance. The add-on of animal-assistance in psychotherapy might be especially helpful for patients that would usually not participate in a psychotherapeutic treatment or experience stress within the dyadic psychotherapeutic setting. These patients therefore are at a greater risk for a chronic course of the experienced psychological difficulties. Therefore, patients’ characteristics such as premorbid social and interpersonal functioning, attachment-styles and potential developmental trauma should be assessed independently of somatic comorbidities. This will help to investigate for whom an animal-assisted approach is superior to a standard psychotherapeutic intervention and support developments of guidelines for differential indications to ensure a maximization of the cost-effectiveness of animal-assisted interventions.

## Conclusion

Patients with acquired brain injury who received the MSCBI experienced a decrease in general psychological distress with and without animal-assistance. Animal-assistance was associated with a higher improvement in patients’ emotional states within-sessions regarding feeling secure, accepted, comforted, motivated, grateful and at ease during the therapy sessions. Animal-assistance also seemed to enhance adherence to treatment. These results support the feasibility of an animal-assisted MSCBI for patients with acquired brain injury and indicate that integrating animals might increase acceptability and patients’ commitment to a psychotherapeutic intervention.

## Methods

### Study design

The presented study was designed as a randomized controlled trial conducted at a neurorehabilitation clinic in Switzerland (REHAB Basel). Patients were randomly assigned to either receive the animal-assisted MSCBI or the standard MSCBI. Both groups had access to treatment as usual and were followed-up until four weeks after completing the intervention. The study was registered at ClinicalTrials.gov (Identifier: NCT03729908, 05/11/2018) and approved by the Ethics Committee for Northwest and Central Switzerland (2018-00564). All research was performed in accordance with relevant guidelines and regulations and the Declaration of Helsinki. The animal-related protocols were approved by the animal ethics board of the Veterinary Office of the Canton Basel-Stadt, Switzerland. The research was conducted in accordance with relevant guidelines such as the ARRIVE guidelines and the IAHAIO white paper^66^.

### Participants

Thirty-one neurorehabilitation inpatients with an acquired brain injury were recruited from June 2018 to June 2019 and data was collected until September 2019. For inclusion, patients had to meet the following criteria: (a) inpatients of REHAB Basel, (b) diagnosed with an acquired brain injury, (c) achieving a score of ≥ 20 in the Montreal Cognitive Assessment screening tool^67^ (MoCA), (d) experiencing depressive and/or anxiety symptoms and/or problems with psychological adaptation to the injury, (e) willing to work with animals, and (f) German speaking. Psychotherapists, physicians, or neuropsychologists proposed inpatients for the study. Patients were then screened for inclusion criteria. If patients met all inclusion criteria, they were informed about the procedures. All patients provided written informed consent.

### Procedure

As indicated in Fig. 1, thirty-one patients were randomly assigned to either the group with animal-assistance (animal-assisted MSCBI: *n* = 14) or without animal-assistance (standard MSCBI: *n* = 17). Randomization was stratified by cognitive status (MoCA-score) and age. Cognitive status was divided into three groups according to the MoCA-score: ≥ 26: normal cognitive functioning, 24–26: marginal cognitive impairment, 20–23: slight cognitive impairment^67,68^. Random numbers were generated with Microsoft Excel and randomization was performed blind by a study-independent researcher.

### Intervention

Table 5 summarizes the intervention protocol and the contents of each session. The psychotherapeutic intervention was designed for a group of 3 to 5 patients. It included main components of the Mindfulness-Based Compassionate Living^69^ (MBCL), the Mindfulness-Based Stress Reduction^70^ (MBSR) and the Mindfulness-Based Cognitive Therapy^71^ (MBCT) program and was supplemented with relational mindfulness- and attachment-focused aspects^72^. Both groups completed the identical manualized treatment protocol. Patients with acquired brain injuries have problems with memory, information processing, orientation, understanding verbal instructions, attention and concentration. To account for these difficulties, each session was highly structured and contained repetitive elements to enhance predictability of the sessions’ contents. As attention and concentration difficulties in brain injured patients are very common, the duration of the sessions was shortened from around 120 minutes in standard protocols^55^ to 75 minutes in this MSCBI. Therefore, we increased the total number of sessions. To reduce complexity, the content of the modules was simplified using easy language and supporting illustrations. After every session, the patients received a memory card and a recording for home-practice. To be able to integrate the techniques in daily life, patients were encouraged to integrate home-practice in their weekly schedule in consultation with the responsible nursing professional at the clinic. The animal-assisted MSCBI and the standard MSCBI were performed by different psychotherapists to account for therapist allegiance regarding animal-assistance^73^.

**Table 5:** Content of the six modules of the mindfulness- and self-compassion-based psychotherapeutic group intervention with (AA-MSCBI) and without (MSCBI) animal-assistance for patients with acquired brain injury.

Module Sessions	Themes	Contents	AA-MSCBI	Exercises	Daily homework for the following week
1 1,2	Basic aspects of mindfulness and self-compassion	1 [Organizational aspects in terms of the intervention. Introduction to the concept of mindfulness, underlining the interconnectedness of thoughts, emotions and physical sensations. Tale illustrating the core concepts of mindfulness]	Minipigs	Mindful hearing meditation 5 Senses raisin exercise Exercise focusing on the tactile sense	Daily timeout
		2 [Introduction to the concept of self-compassion. Tale illustrating the core concepts of self-compassion]	Sheep	Mindful hearing meditation Imagination of a significant other	
2 3,4	Mindful awareness of physical sensations regarding stress and relaxation Attitude of self-compassion	3 [Awareness of stress and accompanied physical sensations]	Minipigs	Mindful hearing meditation Progressive muscle relaxation	Progressive muscle relaxation Mindful breathing exercise
		4 [Awareness of physical sensations of relaxation. Attitude of self-compassion]	Horses	Mindful hearing meditation Mindful breathing excersise	
3 5,6	Mindful awareness of thoughts and behavioral impulses Attitude of self-compassion	5 [Awareness of thoughts. Identifying automatic thoughts]	Goats	Mindful hearing meditation Thought-provoking exercise “Letting go of thoughts” exercise	Body scan
		6 [Awareness of behavioral impulses. Attitude of self-compassion]	Horses	Mindful hearing meditation Awareness of thoughts and behavioral impulses Fostering self-compassion when experiencing difficult thoughts Body scan	
4 7,8	Mindful awareness of emotions and avoidance behavior Attitude of self-compassion	7 [Awareness of emotions. Awareness of approach–avoidance behavior]	Goats Sheep Minipigs	Mindful hearing meditation Approach–avoidance exercise	Loving kindness meditation
		8 [Attitude of self-compassion when confronted with difficult emotions and avoidance tendencies]	Horses	Mindful hearing meditation How to do something good to ourselves Loving kindness meditation	
5 9,10	Integration Transferable skills Attitude of self-compassion	9 [Repetition of the contents of the intervention. Preparing a plan to integrate exercises in daily life]	Goats Sheep Minipigs	Mindful hearing meditation Walking meditation	Integration of homework exercises from the intervention in daily life
		10 [10 Positive emotions of positive psychology. Attitude of self-compassion]	Horses	Mindful hearing meditation Examples of positive emotions in daily life	
6 11,12	Mindfulness in interpersonal relationships Attitude of self-compassion	11 [Mindful interactions. Perceiving one’s boundaries in the interpersonal context]	Goats Sheep Minipigs	Mindful hearing meditation Proximity–distance exercise	Integration of homework exercises from the intervention in daily life
		12 [Retrospection of the intervention. Saying goodbye to the patients and animals, focusing on gratitude and self-compassion]	Horses	Mindful hearing meditation Writing a compassionate letter to future self	

Every module consisted of 2 sessions per week, resulting in 12 sessions in total.

#### Animal-assisted psychotherapeutic mindfulness- and self-compassion-based group intervention (animal-assisted MSCBI)

The animal-assisted MSCBI was carried out by two psychotherapists with a certificate in animal-assisted therapy and training in mindfulness- and self-compassion-based techniques. The sessions took place either in a room, directly at the stables or outdoors. Involved animals were horses, a mule, minipigs, goats andsheep, which all were active in the different exercises of the modules (see Table 5). Animal-assistance was either active or passive. The *mindful breathing exercise* (Module 2, see Table 5) and the *proximity distance exercise* (Module 6, see Table 5) are examples for active animal-assistance. In the *mindful breathing exercise*, the patients first felt the rhythm of the breath of the horse, then concentrated on their own breath while touching their own abdomen and finally tried to synchronize their breathing rhythm with the slow breathing rhythm of the horse. In the *proximity–distance exercise* patients led the horse in silence while holding the rope tensely, then very loosely and finding a position in the middle, focusing on the bodily sensations the distances evoked. An example for passive animal-assistance was the hearing-meditation performed at the beginning of every session with surrounding sounds of the birds in the aviary and animals surrounding the animal-assisted therapy facilities. After each exercise, the patients discussed about upcoming feelings, thoughts, and physical sensations experienced.

The sessions were performed according to the guidelines of the White Paper of the International Association of Human Animal Interaction Organizations^66^ (IAHAIO) to ensure best practices in delivering animal-assisted therapy including the health and well-being of people and animals involved. All animals were trained, accustomed to working with this group of patients and had the possibility to retreat at any time.

#### Psychotherapeutic mindfulness- and self-compassion-based group intervention (standard MSCBI)

The active control group received the same treatment protocol without animal-assistance (see Table 5). The intervention was carried out by four psychotherapists with training in mindfulness- and self-compassion-based techniques. The standard MSCBI was predominantly held in a room inside the clinic. The walking exercises were held outside or inside the clinic. For the *proximity distance exercise*, patients held their hands together in a dyad. The task consisted of one person leading and one following, to alternate the roles and finally to lead and follow achieving a balance of proximity and distance without defining the roles in silence. The *mindful hearing meditation* at the beginning of each session was performed with everyday sounds inside the clinic. All the exercises were followed by a discussion of upcoming feelings, thoughts, and physical sensations.

### Measures

For the primary outcome, patients completed self-report questionnaires at baseline (t_1_; week 0), post-treatment (t_2_; week 7), and follow-up treatment (t_3_; week 11). For the secondary outcome, patients completed self-report questionnaires regarding their emotional states before and after each session. This paper focuses on general psychological distress as well as emotional states, adherence, and attrition. Other measures collected in this study will be published separately.

#### Primary outcome measure

##### Brief symptom inventory

The primary outcome was the Global Severity Index of the Brief Symptom Inventory^74^, measured as change from pre- (t_1_) to post-treatment (t_2_) and from pre-treatment (t_1_) to follow-up treatment (t_3_). This 53-item self-report questionnaire asks patients to rate items considering the past seven days on a 5-point scale, ranging from *not at all [0]* to *extremely [4]*. It consists of nine subscales (somatization, obsessive–compulsive, interpersonal sensitivity, depression, anxiety, hostility, phobic anxiety, paranoid ideation and psychoticism). The Global Severity Index reflects the general psychological distress of the person**.** The internal consistency for the Global Severity Index in the present sample was Cronbach’s α = 0.97.

#### Secondary outcome measures

##### Visual analogue scale

The within-sessions changes of the patients’ emotional states were measured via seven Visual Analogue Scales (VAS) with semantic differentials assessing characteristics on a bipolar dimension^75^. Patients were asked to evaluate the seven dimensions directly before and after each session with a cross on a line ranging from 0 mm (e.g., *discouraged*) to 160 mm (e.g., *motivated*). The following semantic differentials were used: *thankless–grateful, worried–at ease, hopeless–hopeful, discouraged–motivated, disapproved–accepted, insecure–secure* and *uncheered–comforted.*

##### Adherence to treatment and attrition

Acceptability was measured via adherence to treatment, operationalized by the number of attended sessions. The attrition rate was operationalized by the number of dropouts, assessed for each group separately. Dropouts were classified into two categories: patients that left the study before the start of the intervention or during the intervention.

#### Other measures

##### Demographics

Sociodemographic data, including gender, birth date, marital status, level of education, parallel psychotherapeutic treatment, premorbid psychiatric difficulties, pet ownership and affection to animals (*I like animals: Not true at all [1], does not apply [2], rather does not apply [3], rather applies [4], true [5], completely true [6]*) were measured via questionnaire after randomization and before the start of the intervention.

##### Adherence to treatment protocol

A trainee was present during all the sessions and evaluated therapist’s adherence to the treatment protocol with a checklist.

### Statistical analysis

#### Power analysis

We determined a total of 24 patients via a priori calculations with the software package G*Power. On a basis of two groups, we estimated that a total sample size of *N* = 19 would provide 80%-power at a significance level of 95% to detect a medium effect (*d* = 0.6) and a power of 95% to detect a large effect (*d* = 0.8). In previous studies with mindfulness-based programs for patients with acquired brain injury, medium effect sizes were found^33^. To account for possible loss to post- (t_2_) and follow-up assessment (t_3_), we increased the study sample to *N* = 24. Patients who attended at least nine of twelve sessions of group psychotherapy were classified as completers according to the predefined protocol regarding the primary outcome. In case patients did not attend nine of the sessions and had to be classified as dropouts, we recruited until 24 completers were obtained. This led to a total inclusion of 31 patients.

#### Effects on general psychological distress

A generalized linear model was used to investigate the effect between groups on the change in general psychological distress from pre- to post-treatment (t_1_–t_2_) and from pre- to follow-up treatment (t_1_–t_3_). Timepoints of assessment (t_1_, t_2_, t_3_) acted as a within-subject factor and treatment condition (with or without animal-assistance) as a between-subject factor. Variables were visually checked for normality (histogram and Q-Q-plot). Model diagnostics also included visual checks for normality and homogeneity of residuals. All data were approximately normally distributed. Analyses were based on the intention-to-treat approach. In case of missing data for the primary outcome, we imputed estimated values using the replace missing values command by trend function where missing values are replaced by predicted values.

#### Effects on emotional states

We used generalized linear mixed models to compare the within-sessions changes of the patients’ emotional states between groups. Each VAS semantic differential was tested separately, resulting in seven statistical models. The mixed models included condition (with or without animal-assistance) as fixed factor as well as number of session (from 1 to 12) as repeated measure and a random intercept for subject. The VAS outcome at post-session was used as dependent variable and the respective VAS outcome at pre-session was included as offset. The change of patients’ emotional states within groups was calculated using time (pre-session versus post-session) coded as pre = 0 and post = 1. This leads to a simplification of the interpretation of the pre-post-effect as the coefficient directly shows the effect of the session. The model in total allows for the estimation of the group difference (between group effect) and for in-session changes within each group. The results are presented as change scores (post minus pre) for their simpler interpretation. All variables were visually checked for normality (histogram and Q-Q-plot). Model diagnostics included visual checks for normality and homogeneity of residuals. All data were approximately normally distributed. No data were excluded except from missing values (no imputation). If a patient was categorized as dropout regarding the primary outcome because she/he attended less than 9 sessions, available data until the last attended session was included for the in-session analyses regarding the secondary outcome.

Results are analyzed and reported according to the CONSORT 2010 statement^76^. Data are presented as means and standard deviations. For all analyses, the mean difference (*difference*) was used as effect size, the confidence interval was defined at 95% and the significance level was set at 0.05. All statistical analyses were performed with the Statistical Package for Social Science, Version 27 (IBM SPSS® Statistics).

## Supplementary Information

Supplementary Information.

## Data Availability

The datasets generated and analyzed during the current study are available in the Harvard Dataverse repository [https://doi.org/10.7910/DVN/QPRCDJ].
