# Publisher Correction: Effects of animal-assisted psychotherapy incorporating mindfulness and self-compassion in neurorehabilitation: a randomized controlled feasibility trial

**DOI:** 10.1038/s41598-022-17672-4

**Published:** 2022-08-07

**Authors:** Pascale Künzi, Michael Ackert, Martin grosse Holtforth, Margret Hund-Georgiadis, Karin Hediger

**Affiliations:** 1 Division of Clinical Psychology and Psychotherapy, Faculty of Psychology, University of Bern, Bern, Switzerland; 2 REHAB Basel, Clinic for Neurorehabilitation and Paraplegiology, Basel, Switzerland; 3 Psychosomatic Medicine, Department of Neurology, Inselspital, Bern University Hospital, Bern, Switzerland; 4 Division of Clinical Psychology and Animal-Assisted Interventions, Faculty of Psychology, University of Basel, Basel, Switzerland; 5 Department of Epidemiology and Public Health, Human and Animal Health Unit, Swiss Tropical and Public Health Institute, Allschwil, Switzerland; 6 Institute for Interdisciplinary Research on Human-Animal Interaction (IEMT), Allschwil, Switzerland; 7 Department of Psychology, University of Fribourg, Fribourg, Switzerland; 8 Faculty of Psychology, Open University, Heerlen, The Netherlands

Correction to: *Scientific Reports* 10.1038/s41598-022-14584-1, published online 28 June 2022

The original version of this Article contained an error in Table 1, where the value ‘Not specified: missing^a^’ was omitted under the subheading ‘Premorbid psychological difficulties, *N* (%)’. The correct and incorrect values appear below.

Incorrect:

**Table:** 

**Premorbid psychological difficulties, *N* (%)**	
XXX	

Correct:

**Table:** 

**Premorbid psychological difficulties, *N* (%)**	
Not specified: missing^a^	

The original article has been corrected.

