# Supplementary material for: Effects of animal-assisted psychotherapy incorporating mindfulness and self-compassion in neurorehabilitation: a randomized controlled feasibility trial
Source: Sci Rep. 2022 Jun 28;12:10898. doi: 10.1038/s41598-022-14584-1 (PMC9240064; doi:10.1038/s41598-022-14584-1)
Supplement: Supplementary file 1 — Supplementary Information. [file 41598_2022_14584_MOESM1_ESM.pdf]

Supplementary Material for:
Effects of animal-assisted psychotherapy incorporating mindfulness and self-compassion in neurorehabilitation: A randomized controlled feasibility trial

Pascale Künzi*[1,2,6], Michael Ackert[7], Martin grosse Holtforth[1,3], Margret Hund-Georgiadis[2], Karin Hediger[2,4,5,6,8]

1. Division of Clinical Psychology and Psychotherapy, Faculty of Psychology, University of Bern, Switzerland
2. REHAB Basel, Clinic for Neurorehabilitation and Paraplegiology, Basel, Switzerland
3. Psychosomatic Medicine, Department of Neurology, Inselspital, Bern University Hospital, Bern, Switzerland
4. Division of Clinical Psychology and Animal-Assisted Interventions, Faculty of Psychology, University of Basel, Switzerland
5. Department of Epidemiology and Public Health, Human and Animal Health Unit, Swiss Tropical and Public Health Institute Basel, Switzerland
6. Institute for Interdisciplinary Research on Human-Animal Interaction (IEMT), Basel, Switzerland
7. Department of Psychology, University of Fribourg, Switzerland
8. Faculty of Psychology, Open University, Heerlen, the Netherlands

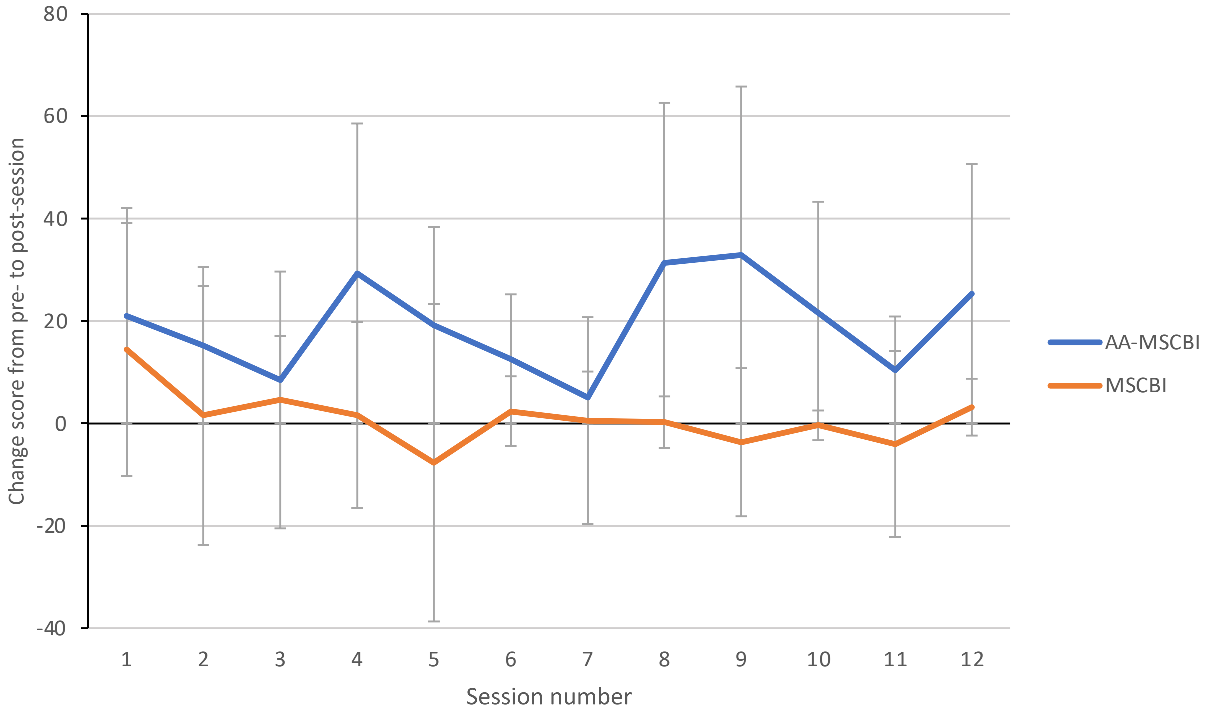

Supplementary Figure 1S. Change in patients' emotional state "secure" from pre- to post-sessions over time (session 1-12) for the animal-assisted psychotherapeutic mindfulness- and self-compassion-based group intervention (AA-MSCBI) and the standard psychotherapeutic mindfulness- and self-compassion-based group intervention (MSCBI). A positive change score indicates an increase, a negative change score indicates a decrease in the mentioned emotional state from pre- to post-session.

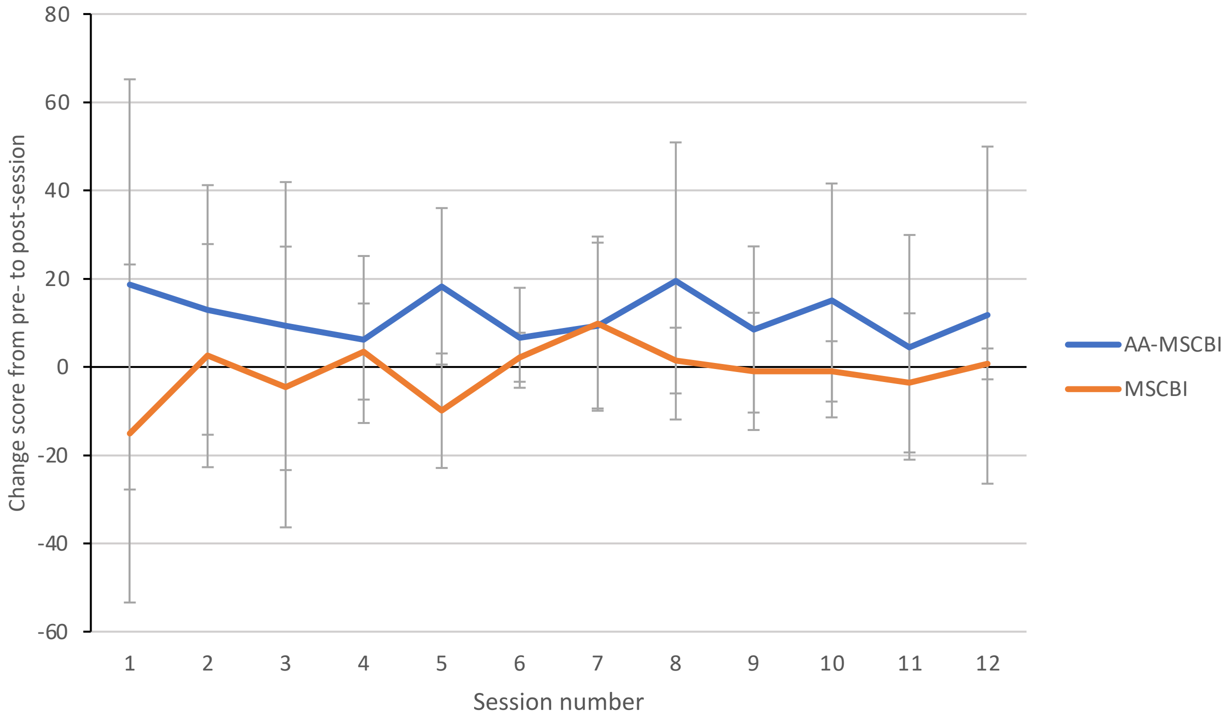

Supplementary Figure 2S. Change in patients' emotional state "comforted" from pre- to post-sessions over time (session 1-12) for the animal-assisted psychotherapeutic mindfulness- and self-compassion-based group intervention (AA-MSCBI) and the standard psychotherapeutic mindfulness- and self-compassion-based group intervention (MSCBI). A positive change score indicates an increase, a negative change score indicates a decrease in the mentioned emotional state from pre- to post-session.

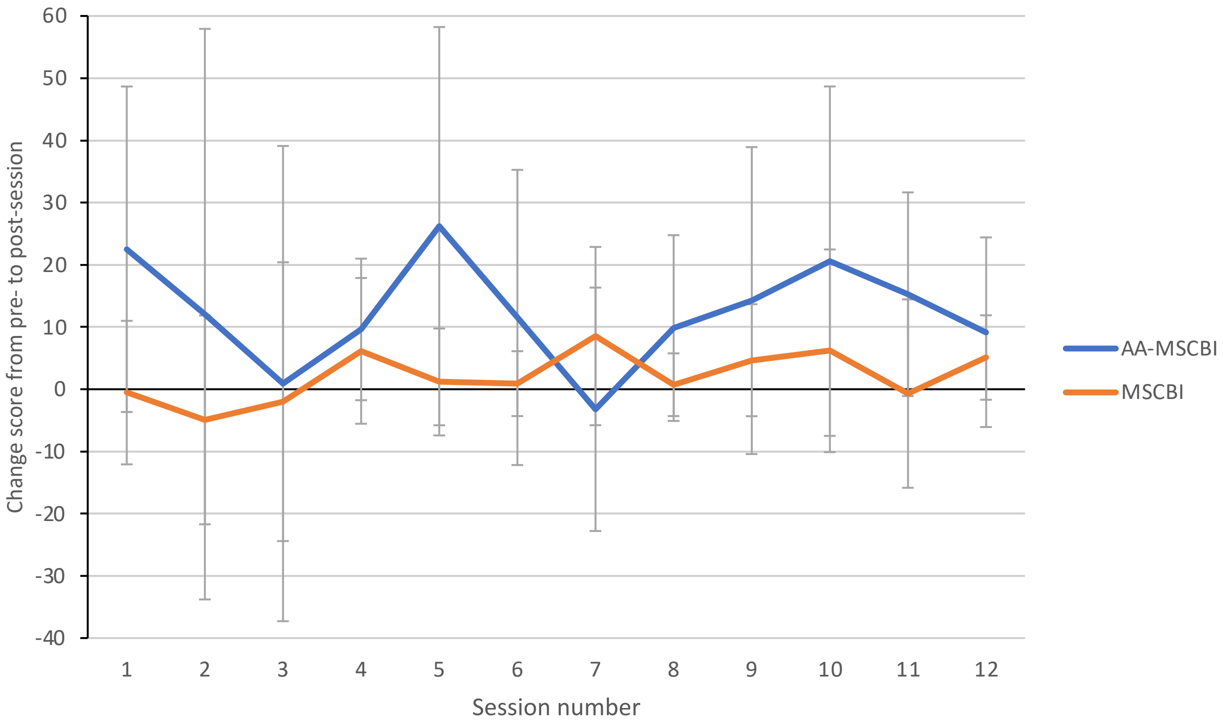

Supplementary Figure 3S. Change in patients' emotional state "accepted" from pre- to post-sessions over time (session 1-12) for the animal-assisted psychotherapeutic mindfulness- and self-compassion-based group intervention (AA-MSCBI) and the standard psychotherapeutic mindfulness- and self-compassion-based group intervention (MSCBI). A positive change score indicates an increase, a negative change score indicates a decrease in the mentioned emotional state from pre- to post-session.

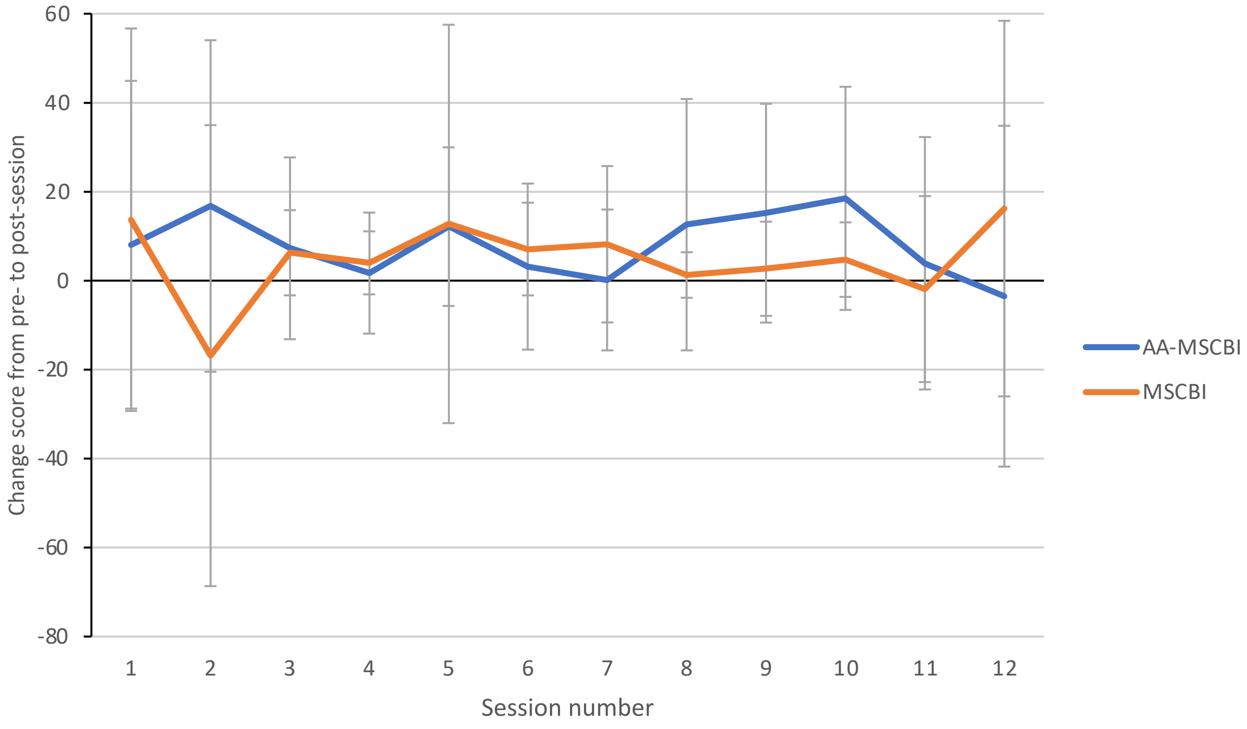

Supplementary Figure 4S. Change in patients' emotional state "hopeful" from pre- to post-sessions over time (session 1-12) for the animal-assisted psychotherapeutic mindfulness- and self-compassion-based group intervention (AA-MSCBI) and the standard psychotherapeutic mindfulness- and self-compassion-based group intervention (MSCBI). A positive change score indicates an increase, a negative change score indicates a decrease in the mentioned emotional state from pre- to post-session.

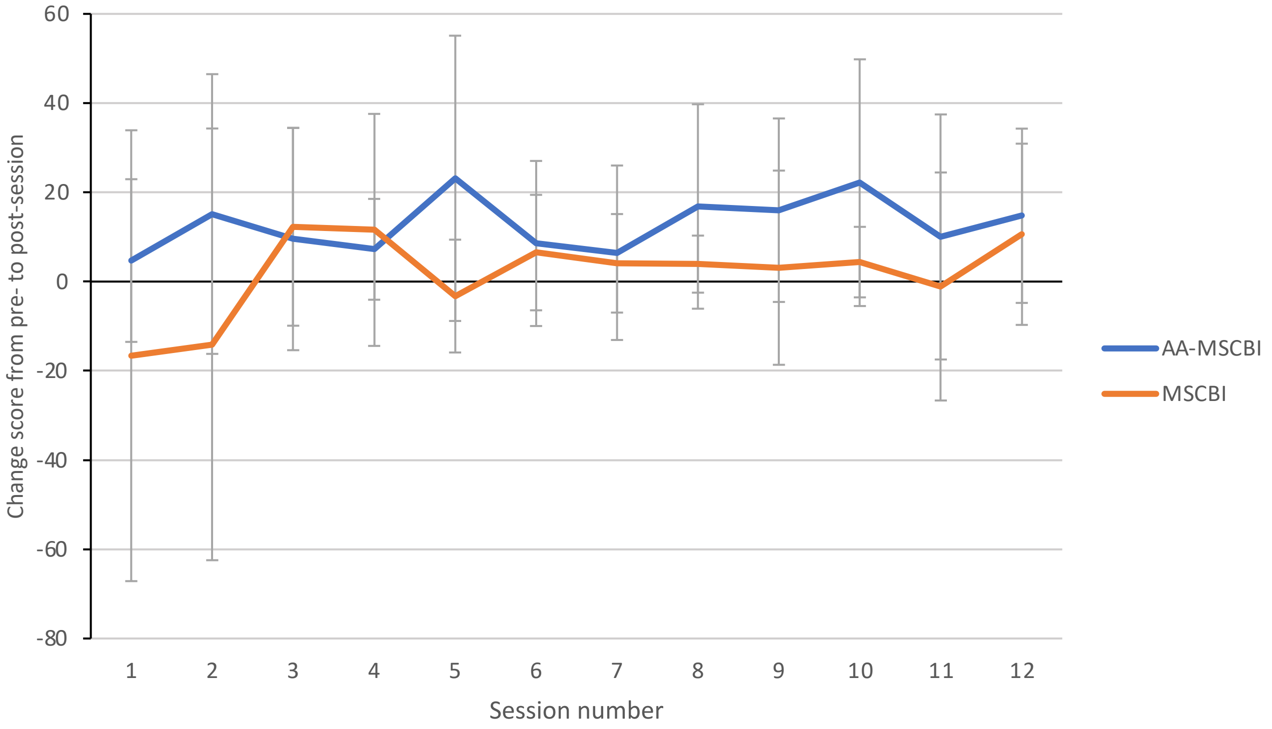

Supplementary Figure 5S. Change in patients' emotional state "motivated" from pre- to post-sessions over time (session 1-12) for the animal-assisted psychotherapeutic mindfulness- and self-compassion-based group intervention (AA-MSCBI) and the standard psychotherapeutic mindfulness- and self-compassion-based group intervention (MSCBI). A positive change score indicates an increase, a negative change score indicates a decrease in the mentioned emotional state from pre- to post-session.

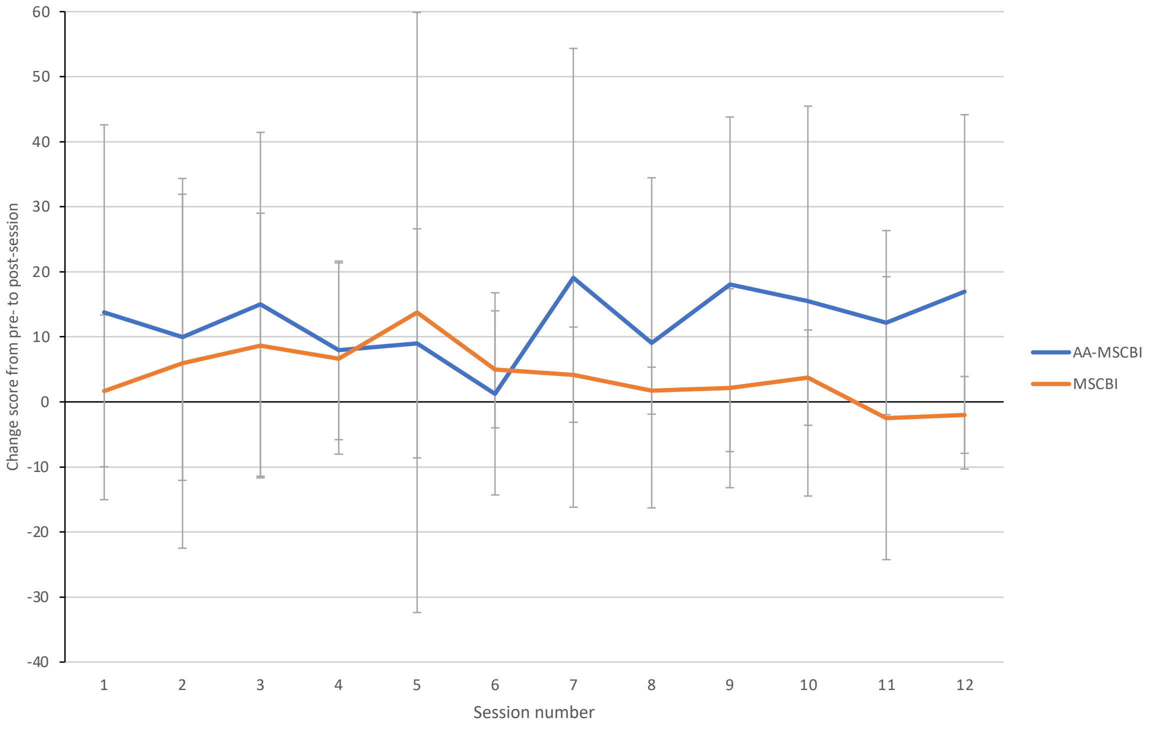

Supplementary Figure 6S. Change in patients' emotional state "grateful" from pre- to post-sessions over time (session 1-12) for the animal-assisted psychotherapeutic mindfulness- and self-compassion-based group intervention (AA-MSCBI) and the standard psychotherapeutic mindfulness- and self-compassion-based group intervention (MSCBI). A positive change score indicates an increase, a negative change score indicates a decrease in the mentioned emotional state from pre- to post-session.

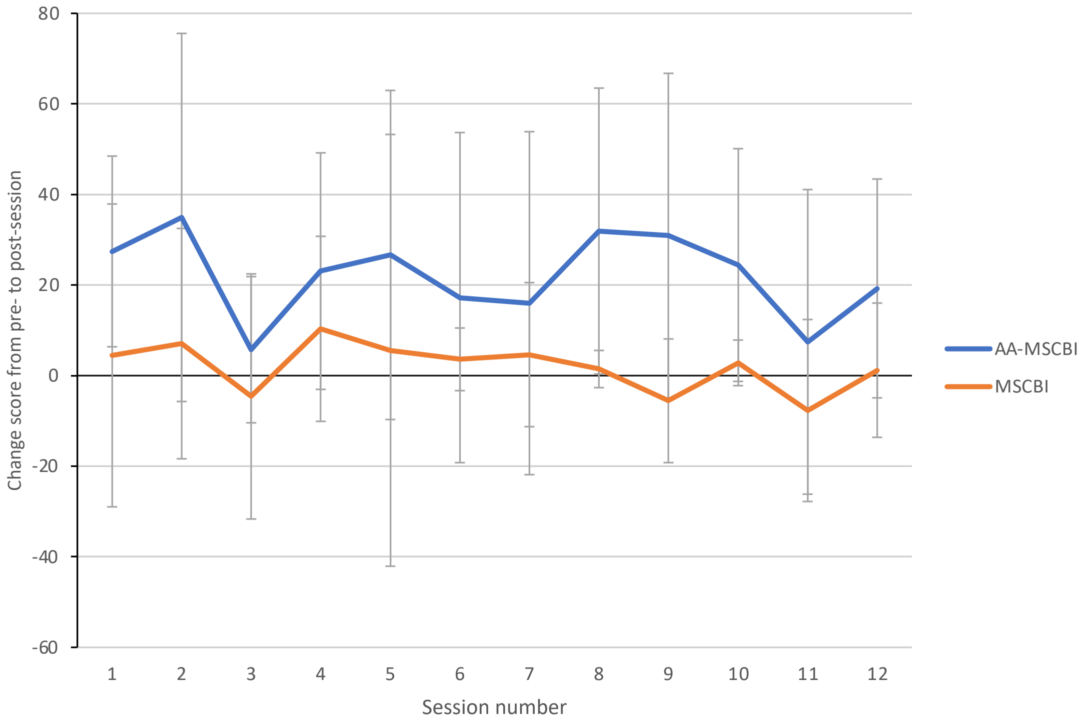

Supplementary Figure 7S. Change in patients' emotional state "at ease" from pre- to post-sessions over time (session 1-12) for the animal-assisted psychotherapeutic mindfulness- and self-compassion-based group intervention (AA-MSCBI) and the standard psychotherapeutic mindfulness- and self-compassion-based group intervention (MSCBI). A positive change score indicates an increase, a negative change score indicates a decrease in the mentioned emotional state from pre- to post-session.
